# Non-Intubated Versus Intubated Video-Assisted Thoracic Surgery in Patients Aged 75 Years and Older: A Propensity Matching Study

**DOI:** 10.3389/fsurg.2022.880007

**Published:** 2022-05-02

**Authors:** Pei-Hsing Chen, Jen-Hao Chuang, Tzu-Pin Lu, Wan-Ting Hung, Hsien-Chi Liao, Tung-Ming Tsai, Mong-Wei Lin, Ke-Cheng Chen, Hsao-Hsun Hsu, Jin-Shing Chen

**Affiliations:** ^1^ Division of Thoracic Surgery, Department of Surgery, National Taiwan University Hospital Yun-Lin Branch, Yunlin County, Taiwan; ^2^ Institute of Biomedical Engineering, College of Medicine and College of Engineering, National Taiwan University, Taipei, Taiwan; ^3^ Department of Surgical Oncology, National Taiwan University Cancer Center, Taipei City, Taiwan; ^4^ Institute of Epidemiology and Preventive Medicine, College of Public Health, National Taiwan University, Taipei City, Taiwan; ^5^ Department of Surgery, National Taiwan University Hospital and National Taiwan University College of Medicine, Taipei City, Taiwan; ^6^ Division of Thoracic Surgery, Department of Surgery, National Taiwan University Hospital and National Taiwan University College of Medicine, Taipei City, Taiwan; ^7^ Graduate Institute of Clinical Medicine, National Taiwan University College of Medicine, Taipei, Taiwan; ^8^ Department of Traumatology, National Taiwan University Hospital, Taipei City, Taiwan

**Keywords:** lung cancer surgery, thoracoscopy/VATS, thoracoscopic surgery, nonintubated surgery, uniportal thoracoscopic surgery, elderly

## Abstract

**Introduction:**

In most developed countries, lung cancer is associated with the highest mortality rate among all cancers. The number of elderly patients with lung cancer is increasing, reflecting the global increase in aging population. Patients with impaired lung or cardiac function are at a high risk during intubated general anesthesia, which may preclude them from surgical lung cancer treatment. We evaluated the safety and survival of non-intubated video-assisted thoracoscopic surgery (VATS) versus those of intubated thoracoscopic surgery for surgical resection for lung cancer in older patients.

**Methods:**

Patients aged ≥75 years who underwent non-intubated and intubated VATS resection with pathologically confirmed non-small cell lung cancer, using a combination of thoracic epidural anesthesia or intercostal nerve block and intra-thoracic vagal block with target-controlled sedation, from January 2011 to December 2019 were included. Ultimately, 79 non-intubated patients were matched to 158 patients based on age, sex, body mass index, family history, comorbidity index, pulmonary function (forced expiratory volume in one second/ forced vital capacity [%]), and disease stage. The endpoints were overall survival and recurrence progression survival.

**Results:**

All patients had malignant lung lesions. Data regarding conversion data and the postoperative result were collected. Both groups had comparable preoperative demographic and cancer staging profiles. The anesthetic duration in the non-intubated group was shorter than that in the intubated group, which showed a significantly higher mean number of lymph nodes harvested (intubated vs non-intubated, 8.3 vs. 6.4) and lymph stations dissected (3.0 vs. 2.6). Intensive care unit (ICU) admission rate and postoperative ICU stay were significantly longer in the intubated group. The complication rate was higher and hospital stay were longer in the intubated group, but these differences were not significant (12% vs. 7.6%; *p* = .07, respectively).

**Conclusions:**

In the elderly, non-intubated thoracoscopic surgery provides similar survival results as the intubated approach, although fewer lymph nodes are harvested. Non-intubated surgery may serve as an alternative to intubated general anesthesia in managing lung cancer in carefully selected elderly patients with a high risk of impaired pulmonary and cardiac function.

## Introduction

Due to prolonged human life expectancy and a steady increase in the aged population, the incidence of lung cancer has progressively increased in elderly patients (1, 2). Surgery is still considered the main curative treatment for lung cancer, even in the elderly (3). Older patients are more obviously affected by general anesthesia, resulting in alveolar barotrauma, volutrauma, atelectrauma, and hemodynamic instability in the perioperative period. Elderly patients generally have a poor cardiopulmonary function and a high possibility of decreased forced expiratory volume in one second (FEV1) and FEV1/forced vital capacity (FVC) ratio. Some elderly patients may be treated conservatively because of impaired pulmonary function and a relatively high incidence of cardiopulmonary complications.

Fortunately, rapid improvements in video-assisted thoracic surgery (VATS), including single-port thoracoscopic surgery (4, 5), preoperative localization (6), and the non-intubated method, have extended the surgical indication to include elderly patients. Non-intubated thoracoscopic surgery helps prevent ventilator-induced damage and consequently ventilation-perfusion (V/Q) mismatch and preserves functional residual capacity (FRC), which may decrease due to neuromuscular blockade and lack of spontaneous diaphragmatic contraction (7, 8).

In a previous study, we have proven the feasibility and safety of non-intubated thoracoscopic procedures in geriatric patients (9). Although perioperative outcomes are good, some studies have reported that non-intubated VATS might have the disadvantage of fewer resected lymph nodes compared with the intubated procedure (10). The influence of the operative method in oncological outcomes, especially in the elderly, however, remain unclear. The aim of this propensity-matched study was to discuss the oncological outcomes of non-intubated versus intubated thoracoscopic surgery for lung cancer in elderly patients (age ≥75 years).

## Materials and Methods

### Design, Setting, and Participants

This retrospective case-control study included 4420 patients who underwent pulmonary resection for non-small cell lung cancer (NSCLC) by a single surgical team at our institute between January 2011 and December 2019. Excluded from the study were patients <75 years of age, those with metastatic cancer (stage IV), those who did not intend to undergo curative treatment, and those with incomplete demographic data. Patients suitable for non-intubated VATS need to have a body mass index (BMI) of <25 kg/m^2^ and an American Society of Anesthesiologists Physical Status Classification score of <4. Patients who had prior thoracic surgery or those with difficult airway management were not included in the non-intubated approach.

The patients were compared based on the endpoints of overall survival and recurrence progression survival. Treatment response was evaluated according to the Response Evaluation Criteria in Solid Tumors (version 1.1) and based on systemic imaging, including positron emission tomography/computed tomography (CT), whole-body bone scan, brain/chest/abdominal contrast-enhanced CT, and brain magnetic resonance imaging.

This study was approved by the National Taiwan University Hospital Research Ethics Committee (approval number: 202110074RINA). The requirement for written informed consent was waived because of the retrospective nature of the study.

### Matching and Statistical Analyses

**Figure 1:**
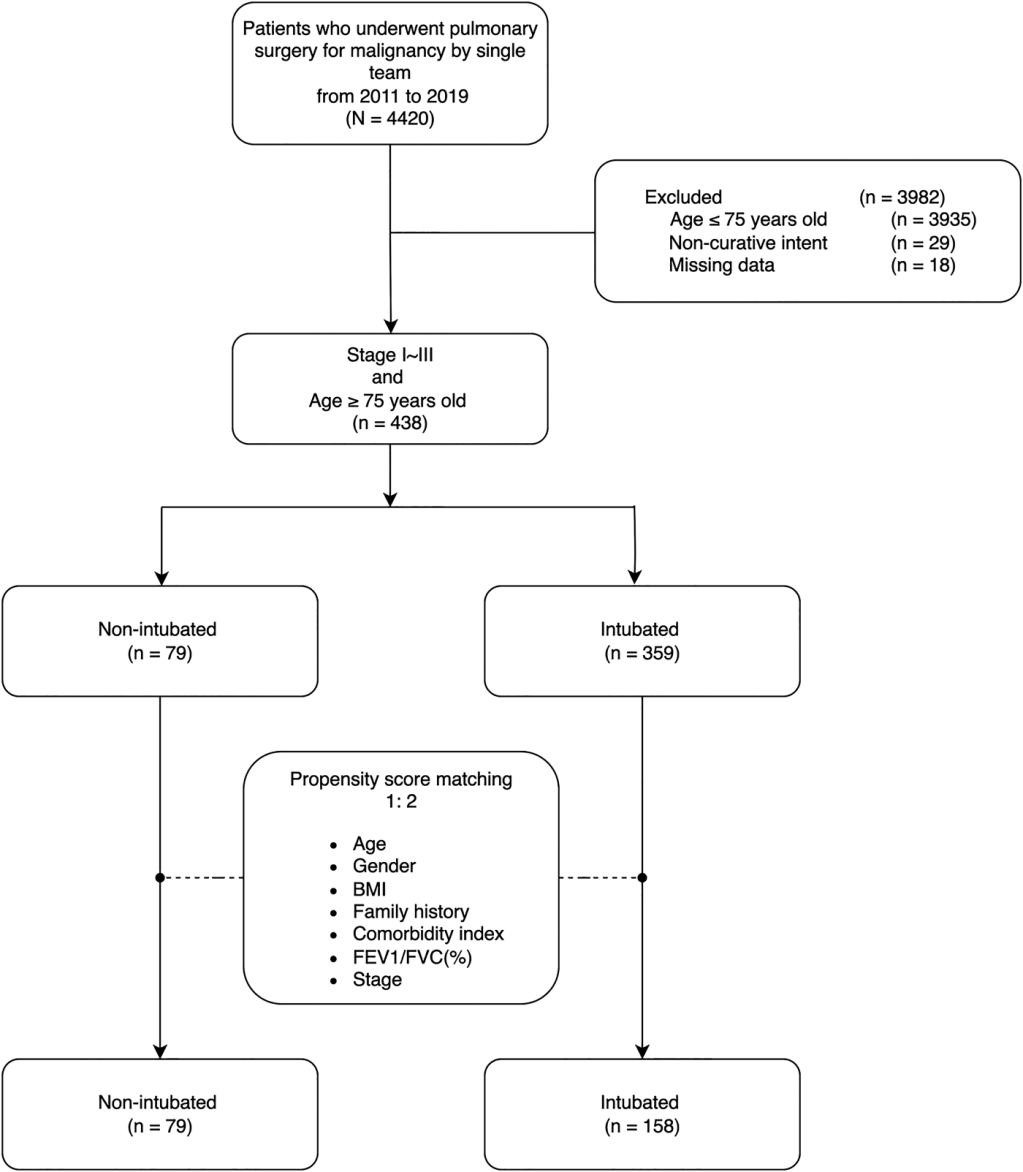
Algorithm for patient selection. Abbreviations: BMI, body mass index; FEV1/FVC, forced expiratory volume in 1 s/forced vital capacity ratio.

A total of 79 non-intubated and 359 intubated patients with stage I to stage III lung cancer according to the International Union Against Cancer/American Joint Committee on Cancer (8th edition) criteria were selected. To reduce selection bias, potential statistically significant (*p* < 0.3) predictors that were closely related to the non-intubated procedure were included. We matched patients with respect to age, sex, BMI, family history, comorbidity index, pulmonary function (FEV1/FVC[%]), and disease stage using the propensity score matching method (caliper width 0.1 of the standard deviation of the propensity score). The matching used a 1:2 ratio greedy (nearest-neighbor) approach with a maximum propensity score difference of ±1%. We calculated the standardized mean difference for each matched variable to check the success of propensity score matching. Ultimately, 79 non-intubated patients were matched to 158 patients. In total, 201 patients were excluded in the control group (Figure 1).

Categorical variable differences between the non-intubated and intubated groups were assessed using the chi-square or Fisher’s exact test. Survival was analyzed using the log-rank test and the Kaplan–Meier method. Complications were documented for each patient according to the Common Terminology Criteria for Adverse Events version 5.0. All comparisons were two-tailed, and a *p*-value of <.05 was considered significantly different. Cox proportional hazards regression analysis was used to calculate hazard ratios (HRs) with 95% confidence intervals.

Statistical analyses were performed using the SPSS software (version 22.0; IBM Corp., Armonk, NY, USA), R (version 3.4.3; R Foundation for Statistical Computing, Vienna, Austria), and R Studio (version 1.1.414; RStudio, PBC, Boston, MA, USA).

### Anesthesia in Non-Intubated Patients

The anesthesia techniques used herein have been described previously (9, 11, 12). Sedation was performed using intravenous administration of propofol (10 mg/mL) followed by intravenous fentanyl (0.5–2 µg/kg) or rocuronium (0.6 mg/kg). Standard monitors included electrocardiograms, along with those that displayed pulse oximetry data, arterial blood pressure, body temperature, and urine output. The end-tidal carbon dioxide and respiratory rate were evaluated using a detector that was placed into one nostril or in front of the mouth. The sedation level was monitored using the frontal bispectral index (BIS Quatro; Aspect Medical System, Norwood, MA, USA) or the Ramsay sedation score. Sedation was maintained at a Ramsay sedation score of II or a bispectral index value of 40–60. A ventilation mask or high-flow nasal cannula was applied to maintain the patient’s oxygen saturation at <90% during the intraoperative period.

Regional anesthesia was administered using the thoracic epidural or intercostal blocks. The T5/T6 thoracic interspace was the target area for epidural catheter insertion to achieve a sensory block with target dermatomes from T2 to T9 with infusion of 2% lidocaine.

### Anesthesia in Intubated Patients

The monitor system was similar as that for the non-intubated patients (9, 11). Anesthesia was induced by the intravenous administration of the propofol, thiopental (3–5 mg/kg), rocuronium (0.6 mg/kg), or fentanyl. Sevoflurane and rocuronium were used for anesthesia maintenance. A standard left-sided double-lumen endotracheal tube was inserted for ventilation. Regional anesthesia was also administered in intubated patients. Patients were extubated immediately after surgery or in the intensive care unit.

## Results

### Participant Characteristics

The data of the 79 patients who underwent non-intubated surgery were compared to those of the 158 matched patients who underwent intubated surgery. **Table 1** shows the demographic data before and after matching. The baseline characteristics were well balanced between the groups. Potential group differences were observed in age, BMI, family history, Charlson comorbidity index, pulmonary function (FVC), and stage before matching (**Table 1**). No differences were observed in the demographic data of the two groups after matching. The mean age of all the matched 237 patients was 79.3 years (79.3 ± 3.6 and 79.4 ± 3.7 for the intubated group and non-intubated group, respectively). A majority of the patients had an Eastern Cooperative Oncology Group performance status of 0 (intubated group, 76.3%, non-intubated group, 84.2%; female: intubated group, 54.4%, non-intubated group, 58.2%) and had early-stage disease (intubated group, 86.7%, non-intubated group, 87.3%). The mean comorbidity index was 5.9 for both groups.

**Table 1:** Demographic and clinical features after propensity score matching.

	Before matching			After matching		
	Intubated	Non-intubated	*p*-value	Intubated	Non-intubated	*p*-value
	(*n* = 359)	(*n* = 79)		(*n* = 158)	(*n* = 79)	
Age, years	78.5 ± 3.2	79.4 ± 3.7	.05	79.3 ± 3.6	79.4 ± 3.7	.84
Sex, Male	158 (44.0)	33 (41.8)	.72	72 (45.6)	33 (41.8)	.58
BMI	24.5 ± 3.6	21.9 ± 2.7	<.001	22.1 ± 2.7	21.9 ± 2.7	.48
Smoker	80 (22.3)	19 (24.1)	.73	35 (22.2)	19 (24.1)	.74
Family history	37 (10.3)	13 (16.5)	.12	22 (13.9)	13 (16.5)	.61
ECOG^a^			.33			.18
0	232 (64.6)	64 (81.0)		100 (63.2)	64 (81.0)	
≥1	61 (17.0)	12 (15.2)		31 (19.6)	12 (15.2)	
Comorbidity index (CCI)	5.8 ± 1.1	5.9 ± 1.1	.25	5.9 ± 1.2	5.9 ± 1.1	.78
PFT						
FVC, %	107.3 ± 18.2	112.2 ± 19.3	.03	111.5 ± 19.0	112.2 ± 19.3	.80
FEV1, %	113.6 ± 26.8	118.5 ± 28.2	.14	116.8 ± 28.0	118.5 ± 28.2	.65
Stage			.16			.97
I	281 (78.3)	69 (87.3)		137 (86.7)	69 (87.3)	
II	38 (10.6)	6 (7.6)		13 (8.2)	6 (7.6)	
III	40 (11.1)	4 (5.1)		8 (5.1)	4 (5.1)	

*Data are presented as mean ± SD or number (%).*

*BMI, body mass index; ECOG, Eastern Cooperative Oncology Group performance status; PFT, pulmonary function test; FEV1, forced expiratory volume in 1 s; FVC, forced vital capacity; CCI, Charlson comorbidity index.*

*^a^* *The Eastern Cooperative Oncology Group performance status of 75 patients was not recorded and was not measured in the analysis.*

### Perioperative Outcomes and Long-Term Survival

Perioperative outcomes after matching are summarized in **Table 2**. All surgeries were performed via video-assisted thoracoscopy. The operation time was longer in the intubated group, but the difference was not significant. The percentage of lobectomies was higher in the intubated group than in the non-intubated group. No conversion in the operative method or from non-intubation to intubation was observed. No 30-day mortality was reported. The intubated group showed a significantly higher mean number of lymph nodes harvested (8.3 vs. 6.4) and lymph stations dissected (3.0 vs. 2.6). Intensive care unit (ICU) admission rate and postoperative ICU stay were significantly longer in the intubated group. The complication rate was higher in the intubated group, but the difference was not significant (12% vs. 7.6%). The hospital stays tended to be longer in the intubated group but not significant (*p* = .07).

**Table 2:** Perioperative outcomes after propensity score matching.

	Intubated	Non-intubated	*p*-value
	(*n* = 158)	(*n* = 79)	
VATS approach	158 (100)	79 (100)	>.99
Operation method			<.001
Lobectomy	58 (36.7)	13 (16.5)	
Segmentectomy	30 (19.0)	16 (20.3)	
Wedge	70 (44.3)	50 (63.3)	
Operative time, min	105.8 ± 54.8	97.5 ± 39.7	.25
Operative bleeding, mL	21.2 ± 77.1	15.2 ± 49.6	.53
Postoperative hospital stay, days	6.6 ± 8.5	4.8 ± 4.2	.07
LN total number	8.3 ± 7.0	6.4 ± 5.6	.04
LN station	3.0 ± 1.8	2.6 ± 1.6	.07
ICU admission	75 (47.5)	24 (30.4)	.01
Postoperative ICU stay, days	0.8 ± 1.5	0.4 ± 0.7	.01
Chest tube			
Chest tube duration, days	3.4 ± 3.9	2.8 ± 4.0	.33
Postoperative complications			
All complications	19 (12.0)	6 (7.6)	.30
Grade II or greater	8 (5.1)	1 (1.3)	.28
Conversion to thoracotomy	0	0	>.99
30-day mortality	0	0	>.99

*Data are presented as mean ± SD or number (%).*

*ICU, intensive care unit; LN, lymph node; VATS, video-assisted thoracic surgery.*

The pathology results for both groups are presented in **Table 3**. The groups were similar in terms of histologic cancer cell type, pathological tumor stage, pathological nodal stage, and resection margin involvement. The pathological features, including differentiation, lymphovascular invasion, and visceral pleural invasion, also showed no significant differences.

**Table 3:** Pathological features after propensity score matching.

	Intubated	Non-intubated	*p*-value
	(*n* = 158)	(*n* = 79)	
Differentiation			.73
Well	29 (18.4)	16 (20.3)	
Moderate poor	129 (81.6)	63 (79.7)	
VPI	50 (31.6)	20 (25.3)	.31
LVI	26 (16.5)	19 (24.1)	.16
Resection margin involvement	12 (7.6)	11 (13.9)	.12
Pathological T stage			.98
I	98 (62.0)	50 (63.3)	
II	49 (31.0)	24 (30.4)	
III	11 (7.0)	5 (6.3)	
Pathological N stage			.68
I	149 (94.3)	76 (96.2)	
II	5 (3.2)	1 (1.3)	
III	4 (2.5)	2 (2.5)	
Histology			.17
Adenocarcinoma	117 (74.1)	66 (83.5)	
SqCC	18 (11.4)	9 (11.4)	
Adenosquamous	17 (10.8)	3 (3.8)	
Pleomorphic	5 (3.2)	0 (0)	
Carcinoid	1 (0.6)	1 (1.3)	

*Data are presented as mean ± SD or number (%).*

*LVI, lymphovascular invasion; VPI, visceral pleural invasion; SqCC, squamous cell carcinoma.*

**Figure 2:**
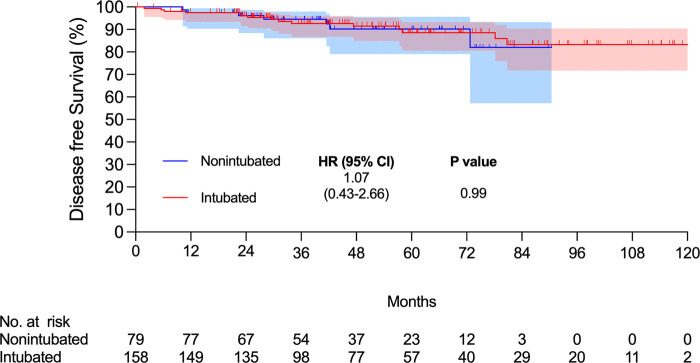
Kaplan–Meier overall survival curve. Abbreviations: CI, confidence interval; HR, hazard ratio.

**Figure 3:**
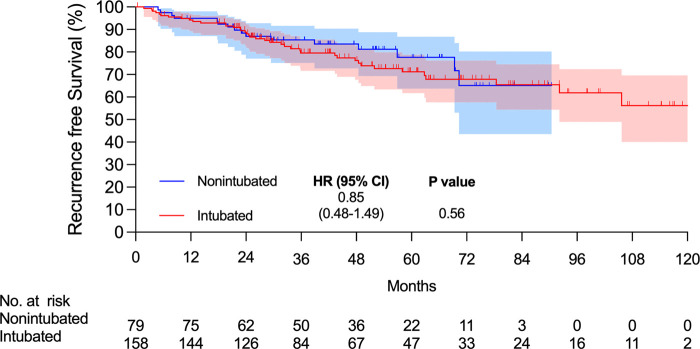
Kaplan–Meier recurrence-free survival curve. Abbreviations: CI, confidence interval; HR, hazard ratio.

There were 15 and 7 mortality events and 40 and 16 recurrences in the intubated and non-intubated groups, respectively. The median follow-up time for all matched patients was 45.5 months. The survival curves are presented in **Figures 2 and 3**. The overall survival and recurrence-free survival (RFS) rates were similar in both groups (*p* = .99 and *p* = .56, respectively).

## Discussion

This propensity-matched case study confirmed that non-intubated thoracoscopic surgery is associated with similar OS and RFS rates in elderly patients aged ≥75 years with pathologically proven NSCLC. Compared to the intubated group, the non-intubated group showed satisfactory perioperative results, as in previous studies (9, 11, 13–15). Although there were fewer lymph nodes harvested in the non-intubated group, it did not influence the long-term results compared to the intubated group. To the best of our knowledge, this is the first report revealing oncologic outcomes in elderly patients with lung cancer undergoing non-intubated thoracoscopic pulmonary resection.

Owing to prolonged life expectancy, lung cancer has become a leading cause of death in the elderly worldwide. Pulmonary resection for lung cancer in elderly patients is still considered an option for first-line treatment. Several studies have demonstrated the feasibility and safety of pulmonary resection in the elderly (16–21). However, this requires careful patient selection and skilled surgery. Fortunately, rapid improvements in thoracoscopy, including single-port surgery (22), preoperative and intraoperative localization techniques (6, 23, 24). wedge resection for subcentimeter lung cancer, and non-intubated thoracoscopic surgery have facilitated pulmonary resection in the elderly. Non-intubated thoracoscopic surgery could be considered a part of enhanced recovery after surgery protocols. It has the benefits of minimizing airway injury, residual neuromuscular blockade, and ventilator-induced lung complications. Elderly individuals are considered to have poor FEV1 and FEV1/FVC ratios compared with the young population. Non-intubated thoracoscopic surgery can help prevent V/Q mismatch and maintain better FRC; it has also extended the criteria for surgery, as opposed to conservative treatment. Advantages such as the faster resumption of postoperative feeding, shorter duration of antibiotic use, shorter hospital stay, and fewer inflammatory responses have been reported for non-intubated thoracoscopic surgery. This may lead to a better recovery course in elderly patients (25, 26).

Our previous report demonstrated the feasibility and safety of non-intubated thoracoscopic surgery in a geriatric population, even when performing lobectomy (9). Although non-intubated thoracoscopic surgery has potential advantages of lower perioperative complications and faster recovery, debates regarding its oncological outcomes and true benefit remain (27). Alghamdi et al. compared 62 patients who underwent VATS lobectomy using the non-intubated and intubated approaches. In that study, although the operative time was shorter in the non-intubated group, the number of harvested lymph nodes in the non-intubated group were significantly fewer than those in the intubated group (13 vs. 18, *p* = .003) (10). In our previous analysis comparing non-intubated uniportal segmentectomy with the intubated approach, the non-intubated group had fewer lymph nodes harvested. This result may be due to the activated cough reflex during non-intubated surgery, which makes it difficult to perform lymph node dissection, especially in male patients with higher BMI (12).

To date, few studies have focused on the survival of patients undergoing non-intubated thoracoscopic surgeries. Wang et al. conducted an analysis of patients with early-stage disease who underwent lobectomy (11). The results showed similar OS and RFS rates for non-intubated and intubated lobectomy. However, to our knowledge, no report has focused on non-intubated thoracoscopic surgery in the geriatric population. Our study also showed no obvious survival benefit in the elderly group; although the number of harvested lymph nodes was fewer in the non-intubated group, the OS and RFS were similar after matching. In addition, perioperative outcomes, such as hospital stay, ICU admission rate, and ICU stay were better in the non-intubated group. The postoperative complication rate was lower for all complications and for complications with a grade >2. Similar survival results in the elderly may support the application of non-intubated thoracoscopic surgery in this subgroup of patients.

To the best of our knowledge, our study is the first to evaluate and compare, using the propensity score matching method, the long-term survival of elderly patients with resectable lung cancer undergoing non-intubated and intubated thoracoscopic surgery. Factors related to the choice of surgical procedure, lymph node dissection, and possible influence on survival, such as age, sex, BMI, family history, pulmonary function, and disease stage, were used to balance the groups. In addition, the comorbidity index was used to ensure that the baseline values in the two groups were equal.

The limitations of this study include its retrospective nature and the fact that the data were collected by a single surgical team. The primary problem in the comparison was selection bias, which we tried to minimize by using propensity score matching to eliminate confounding factors. Moreover, adenocarcinoma was the dominant cell type in the pathology. The recent rapid progression of systemic treatment using epidermal growth factor receptors and anaplastic lymphoma kinase may significantly prolong survival. Our results should be interpreted carefully in populations with other predominant cell types. Further studies should be conducted using a prospective design to reinforce this conclusion.

Despite these limitations, this study showed that non-intubated thoracoscopic surgery provides similar survival results in the elderly as the intubated approach, although fewer lymph nodes were harvested. With preferable perioperative outcomes, non-intubated thoracoscopic surgery may be beneficial in carefully selected elderly patients.

## Data Availability

The original contributions presented in the study are included in the article/Supplementary Material, further inquiries can be directed to the corresponding author/s.
